# Combined effect of Tetracycline compounds and essential oils on antimicrobial resistant *Salmonella enterica* isolated from the swine food chain

**DOI:** 10.3389/fmicb.2024.1439286

**Published:** 2024-12-09

**Authors:** Francesca Maggio, Carlotta Lauteri, Chiara Rossi, Gianluigi Ferri, Annalisa Serio, Alberto Vergara, Antonello Paparella

**Affiliations:** ^1^Department of Bioscience and Technology for Food, Agriculture and Environment, University of Teramo, Teramo, Italy; ^2^Department of Veterinary Medicine, University of Teramo, Piano d’Accio, Teramo, Italy

**Keywords:** antimicrobial resistance, *Salmonella enterica*, essential oils, tetracycline, swine food chain

## Abstract

Antimicrobial resistance (AMR) poses risks for food stakeholders because of the spread of resistant microbes and potential foodborne diseases. In example, pigs may carry *Salmonella* strains, which can infect humans through contaminated food preparations. Due to their antibacterial properties and capacity to modulate bacterial drug resistance, essential oils (EOs) are attracting interest as prospective substitutes for synthetic antimicrobials which can help to reverse microbial resistance. Hence, the present study evaluates the antimicrobial effectiveness of the combination of tetracycline (Tc) compounds and *Coridothymus capitatus* (CC), *Thymus capitatus* L. (TC), and *Thymus serpyllum* (TS) EOs on 11 tetracycline-resistant *Salmonella enterica* strains isolated from the swine food chain. The kind of interaction between Tc and EOs was evaluated by Fractional Inhibitory Concentration Index (FICI), while the composition of the EOs phytocomplex was linked to Tc antibacterial activity by Principal Component Analysis (PCA). Interestingly, the EOs increased the strains susceptibility to Tc, inhibiting their growth despite the antimicrobial resistance. In most cases, synergistic and commutative effects were detected, as the combination of EOs and Tc compounds resulted in a noticeable decrease in the concentration (from 256 to 4 μg/mL) necessary to inhibit the strains. Thymol, carvacrol, linalool, sabinene, and other EO terpenoid components were revealed as the molecules working in concert with the Tc drug to increase the susceptibility of *S. enterica* strains to the treatment. Comprehending which molecules of the EOs phytocomplex, beside the main compounds, affect bacterial inhibition, might help to develop a tailor-made approach related to counteract the resistance of specific strains to different antibiotics.

## 1 Introduction

The livestock production industry raises pigs using a variety of antibiotics to prevent infections and enhance animal health (Magnusson et al., 2019). Nevertheless, ineffective use of antibiotics lowers infection control, raises treatment expenses, and permits the emergence of drug-resistant bacteria, which leads to therapeutic failure (Burow et al., 2019; Jiang et al., 2021). According to Lekagul et al. (2019), tetracycline (Tc) is one of the most commonly used drugs in the production of swine livestock, and genes linked to Tc resistance are among the most prevalent ones found in the swine microbiome. Antibiotics destroy most of the gut microbiota; yet, certain bacteria continue to exist and develop resistance over time, passing on antibiotic resistant genes to the vulnerable microbial community (Wang et al., 2023a; Chen et al., 2020).

A growing number of reports worldwide have documented an increase in the incidence of antimicrobial-resistant *Salmonella* infection, a major public health concern. This suggests that the problem is widespread (EFSA and ECDC, 2022). As documented by several studies (Rapport, 2020; Lauteri et al., 2022a), *Salmonella* spp. strains isolated from the swine production chain are resistant to more than three classes of antibiotic compounds. *Salmonella* spp. outliers revealed a high percentage of Tc resistance, with Italy recording the highest percentage (52.4%) (EFSA and ECDC, 2022). According to Jiang et al. (2021), pigs are considered healthy carriers of *Salmonella* and are the primary source of infection for other animals and environmental contamination. This indicates that people may become infected when swine cross-contaminate other animals through the food chain (Monger et al., 2021). Actually, eating foods produced from animals, such as pigs, beef, lamb, and chicken, is commonly associated with the foodborne illness known as salmonellosis, which is caused by *Salmonella* spp.

Due to their natural origin, low toxicity, and lack of residues, essential oils (EOs) are emerging as potential antibiotic substitutes in this context (Zhang et al., 2022). Pure EOs or their bioactive constituents have been proposed for use in the food supply chain due to the antibacterial activity of the phytocomplex (Speranza et al., 2010). EOs are potent antimicrobials that target several different areas in bacterial cells without causing antimicrobial resistance (Rossi et al., 2022a), at the same time preventing and removing pathogens biofilms from food-contact surfaces (Rossi et al., 2022b). According to Değirmenci and Erkurt (2020), bacteria find it harder to become resistant to EOs than to antibiotics, which are frequently made up of just one chemical entity. For instance, the EOs of *Thymus* and *Origanum* spp. disrupt the membrane’s fatty acid composition, increasing its permeability and compromising energy and respiratory metabolism (He et al., 2022; Lauteri et al., 2022b). As a result, combining antimicrobial compounds with EOs could be a method to increase bacterial susceptibility to antimicrobic treatments. Nevertheless, the question of whether EOs biological effects are represented solely in the primary molecules at the highest concentrations according to the compositional analysis, or if they result from the synergism of all molecules present is still being debated because of the richness and diversity of the whole phytocomplex (Yap et al., 2014).

On this basis, the purpose of this study was to assess the antimicrobial effectiveness of Tc compounds in combination with three EOs, namely *Coridothymus capitatus*, *Thymus capitatus* L., and *Thymus serpyllum*, against 11 strains of *S. enterica* that were previously isolated from the swine food chain and studied by Lauteri et al. (2022b). As an innovative approach, special attention was focused on the role of the EO phytocomplex to boost the antibacterial effectiveness in combination with Tc. Indeed, all the tested EOs were selected for having carvacrol as the main compound and different secondary compounds. This research aimed to investigate the possible correlation between the secondary bioactive components and the overall antibacterial activity, by applying Principal Component Analysis (PCA) as a preliminary approach, also considering the potential strain-specific response. This new viewpoint is expected to give results which might help to relate the presence of particular minor compounds in the EOs to their antimicrobial activity and the interaction with tetracycline in resistant *Salmonella* strains.

## 2 Results

### 2.1 Antimicrobial activity of compounds based on minimum inhibitory concentrations and minimum bactericidal concentrations

Table 1 shows the Minimum Inhibitory Concentrations (MICs) and Minimum Bactericidal Concentrations (MBCs) of EOs and Tc compounds detected after 24 and 48 h at 37°C on the *S. enterica* strains. Among the strains, *Salmonella* Enteritidis ATCC 13076 exhibited susceptibility to treatments, showing no growth even at the lowest concentrations of EOs and Tc compounds (0.156 μL/mL and 4 μg/mL, respectively). Hence, *Salmonella* Enteritidis ATCC 13076 was excluded from further analyses. The MICs and MBCs showed that the *S. enterica* strains were not affected by the highest concentration of Tc compounds used (256 μg/mL). This is in agreement with what Lauteri et al. (2022b) reported about antimicrobial resistance. On the other hand, EOs treatments demonstrated good antimicrobial effectiveness in terms of MICs. Specifically, the growth of the *S. enterica* strains was inhibited within a concentration range of approximately 0.31–5 μL/mL, with TCEO and TSEO exerting antimicrobial effectiveness at lower concentrations.

**Table 1:** MICs and MBCs values of tetracycline (Tc) compounds, *C. capitatus* (CC), *T. capitatus* (TC) and *T. serpyllum* (TS) EOs against the *S. enterica* strains under this study after 24 h and 48 h at 37°C.

Strain	MICs								MBCs							
	24 h				48 h				24 h				48 h			
	CCEO	TCEO	TSEO	Tc	CCEO	TCEO	TSEO	Tc	CCEO	TCEO	TSEO	Tc	CCEO	TCEO	TSEO	Tc
ATCC 13076	<0.156	<0.156	<0.156	<4.00	<0.156	<0.156	<0.156	<4.00	<0.156	<0.156	<0.156	<4.00	<0.156	<0.156	<0.156	<4.00
117	1.25	0.625	1.25	>256	1.25	0.625	1.25	>256	1.25	0.625	1.25	>256	1.25	0.625	1.25	>256
785	0.625	0.625	0.625	>256	0.625	0.625	0.625	>256	0.625	0.625	0.625	>256	0.625	0.625	0.625	>256
783	0.31	0.31	0.31	>256	0.625	0.31	0.625	>256	0.625	0.31	0.625	>256	0.625	0.31	0.625	>256
788	1.25	0.625	2.50	>256	1.25	0.625	2.50	>256	1.25	0.625	2.50	>256	1.25	0.625	2.50	>256
116	0.25	0.625	0.625	>256	5.00	1.25	0.625	>256	0.25	1.25	0.625	>256	5.00	1.25	0.625	>256
208	0.625	1.25	0.31	>256	0.625	1.25	0.31	>256	0.625	1.25	0.31	>256	0.625	1.25	0.31	>256
217	0.25	1.25	1.25	>256	0.25	1.25	1.25	>256	0.25	1.25	1.25	>256	0.25	1.25	1.25	>256
686	5.00	5.00	2.50	>256	5.00	5.00	2.50	>256	5.00	5.00	2.50	>256	5.00	5.00	2.50	>256
787	0.25	1.25	2.50	>256	0.25	2.50	2.50	>256	0.25	1.25	2.50	>256	0.25	2.50	2.50	>256
791	0.25	1.25	2.50	>256	0.25	2.50	2.50	>256	0.25	1.25	2.50	>256	0.25	2.50	2.50	>256
792	1.00	5.00	1.25	>256	1.00	5.00	1.25	>256	1.00	5.00	1.25	>256	1.00	5.00	1.25	>256

MICs and MBCs values are expressed as μL/mL for EOs and μg/mL for Tc. The standard deviation is not reported, being zero for all replicates. EO, *Essential oil*; CC, Coridothymus capitatus; TC, Thymus capitatus; TS, Thymus serpyllum, Tc, Tetracycline compounds. MICs, Minimum Inhibitory Concentrations; MBCs, Minimum Bactericidal Concentrations.

### 2.2 Synergy between the essential oils and the tetracycline compounds

Table 2 shows the results about the combination of EOs and Tc compounds, which caused a clear reduction of the MICs in each *S. enterica* strain. Indeed, the MIC values decreased from 256 μg/mL, as observed in the individual treatment (Table 1), to 4 μg/mL when combined with the EOs, adhering to the susceptibility breakpoints of ≤4 μg/mL outlined in the current Clinical and Laboratory Standards Institute (CLSI) guidelines of 2020 (supplement M100S-Ed30). As shown in Table 2, the MICs and MBCs matched for all tests. The Fractional Inhibitory Concentration Index determination enabled the definition of the resulting activity between the antimicrobial compounds. In detail, the combination between EOs and Tc, presented in Table 2, had a synergistic effect in four out of eleven strains in the presence of CCEO. Additionally, a synergistic effect was observed in three out of eleven strains with TCEO, and finally, in four out of eleven strains with TSEO. Conversely, the combination of EO and Tc demonstrated a commutative effect in two cases in the presence of CCEO, while in one and eight strains out eleven, in TCEO and TSEO, respectively. The indifferent effect was observed only in one case in the presence of CCEO, and in four and seven cases in TSEO and TCEO, respectively. However, antagonistic effects were primarily observed with CCEO in four out of eleven strains. On the other hand, two cases of antagonistic effect were detected in the presence of TSEO, while no cases were observed in presence of TCEO.

**Table 2:** MICs, MBCs, and effects resulting by the combination between *C. capitatus* (CC), *T. capitatus* (TC) and *T. serpyllum* (TS) EOs with tetracycline (Tc) compounds against *S. enterica* strains after 48 h of incubation at 37°C.

	MICs			MBCs			FICI			Effects		
	CCEO-Tc	TCEO-Tc	TSEO-Tc	CCEO-Tc	TCEO-Tc	TSEO-Tc	CCEO-Tc	TCEO-Tc	TSEO-Tc	CCEO-Tc	TCEO-Tc	TSEO-Tc
117	0.31/4	0.31/4	0.31/4	0.31/4	0.31/4	0.31/4	0.2	0.5	0.2	S	S	S
785	1.25/4	0.625/4	0.625/4	1.25/4	0.625/4	0.625/4	2	1	1	C	I	I
783	0.31/4	0.31/4	0.31/4	0.31/4	0.31/4	0.31/4	0.5	1	0.5	S	I	S
788	1.25/4	0.625/4	2.50/4	1.25/4	0.625/4	2.50/4	1	1	0.2	I	I	S
116	0.25/4	0.125/4	0.625/4	0.25/4	1.25/4	0.625/4	0.5	0.1	0.2	S	S	S
208	1.25/4	1.25/4	0.31/4	1.25/4	1.25/4	0.31/4	2	1	4	C	I	A
217	2.50/4	2.50/4	1.25/4	2.50/4	5.00/4	2.50/4	10	2	2	A	C	C
686	0.50/4	2.50/4	5.00/4	0.50/4	2.50/4	5.00/4	0.1	0.5	1	S	S	I
787	2.50/4	2.50/4	2.50/4	2.50/4	2.50/4	2.50/4	10	1	1	A	I	I
791	2.50/4	2.50/4	2.50/4	2.50/4	2.50/4	2.50/4	10	1	1	A	I	I
792	5.00/4	5.00/4	1.25/4	5.00/4	5.00/4	1.25/4	5	1	4	A	I	A

MICs and MBCs values are expressed as μL/mL for EOs and μg/mL for Tc. The standard deviation is not reported, being zero for all replicates. EO, *Essential oil*; CC, Coridothymus capitatus; TC, Thymus capitatus; TS, Thymus serpyllum, Tc, Tetracycline compounds; MICs, Minimum Inhibitory Concentrations; MBCs, Minimum Bactericidal Concentrations; FICI, Fractional Inhibitory Concentration Index. Effects: A, Antagonism; C, Commutative; I, Indifference; S, Synergism. A: FICI value > 2; C: 1 < FICI value ≤ 2; I: FICI value = 1; S: FICI value < 1.

### 2.3 Relationships between the EOs chemical profile and bioactivity of the treatments

**Figure 1:**
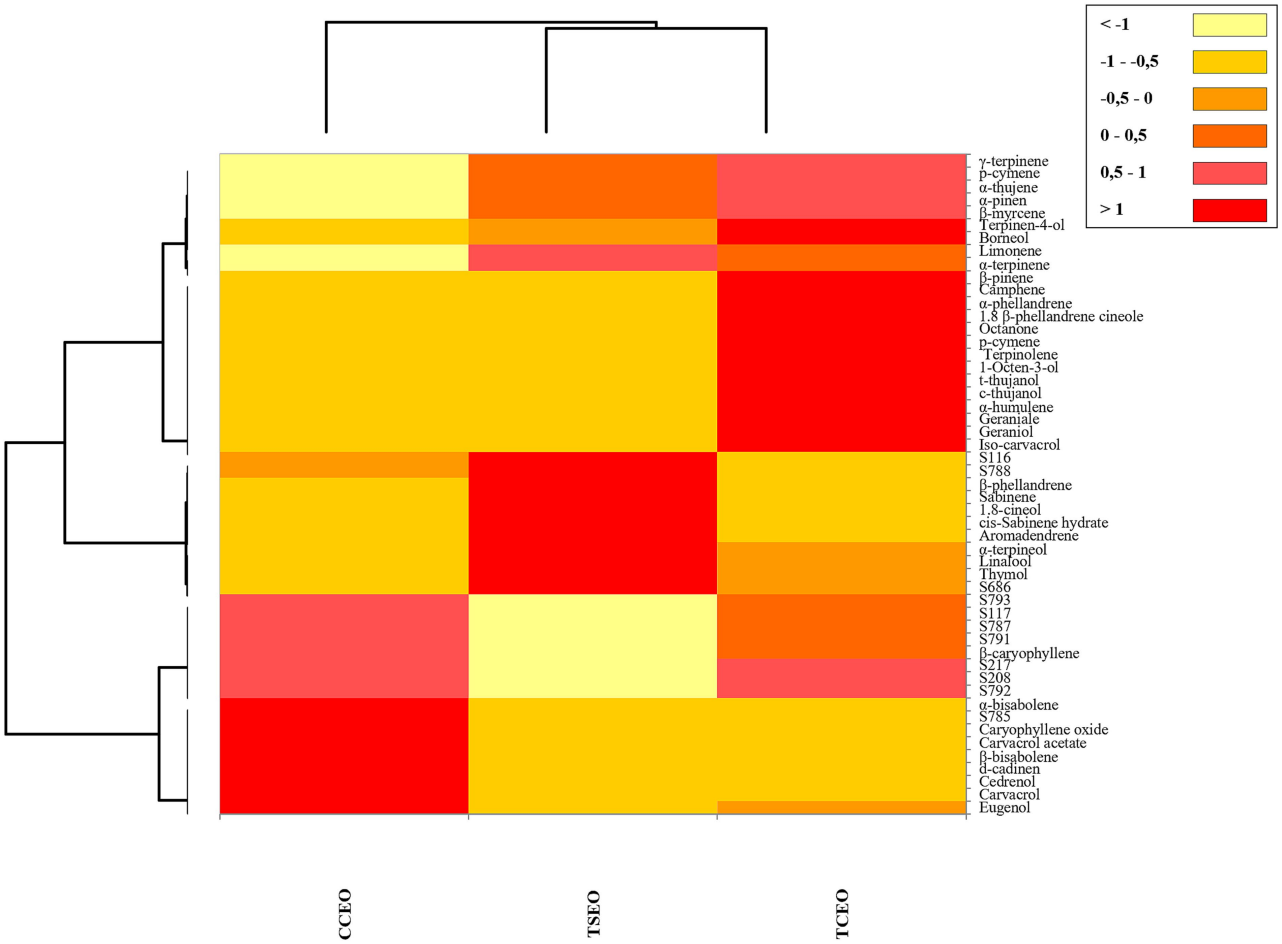
Heatmap diagram based on the fractional volatile compounds amount and on the results of the antibacterial activity (MICs) of *C. capitatus* (CC), *T. capitatus* (TC), and *T. serpyllum* (TS) EOs in combination with tetracycline (Tc) compounds for 48 h at 37°C. Columns represent the three EOs and rows represent the different bioactive compounds and the response of the *S. enterica* strains (S) to treatments. The color intensity is proportional to the amount (μL/mL) of EOs volatile compounds and to the MICs values (μL/mL or μg/mL). The color discoloration starting from red (the highest value) to yellow (the lowest value).

**Figure 2:**
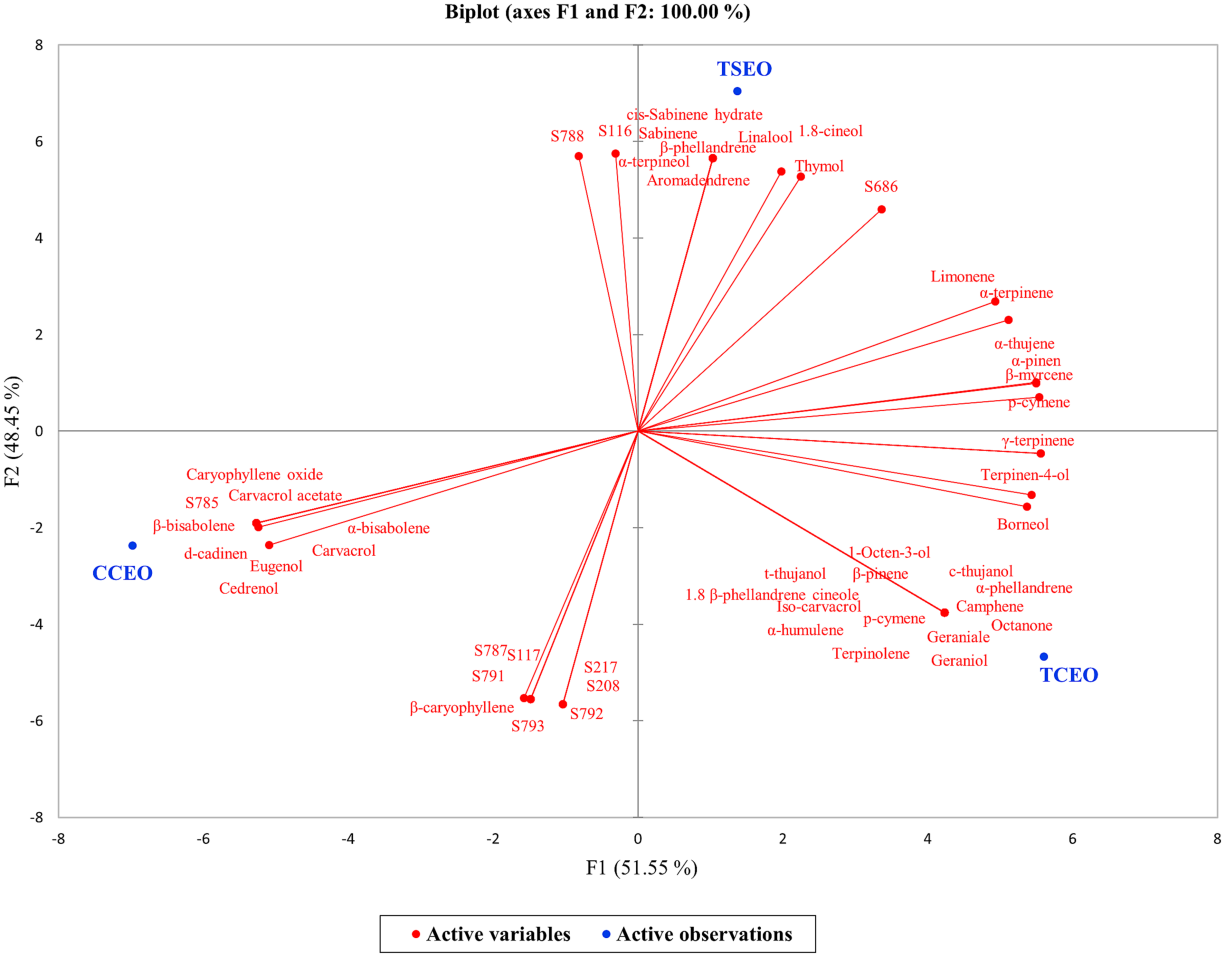
PCA biplot based on antibacterial activity of *C. capitatus* (CC), *T. capitatus* (TC) and *T. serpyllum* (TS) EOs in combination with tetracycline (Tc) compounds at 37°C for 48 h on the *S. enterica* strains (S) under this study.

Figures 1, 2 show the correlation between the antibacterial effectiveness of Tc and the EOs phytocomplex, represented by Heatmap and PCA. In this way, the concentration of volatile compounds in EOs (expressed as μL/mL) and the response in terms of MICs of the *S. enterica* strains exposed to the mix of antimicrobial agents for 48 h at 37°C were linked.

The Heatmap revealed that CCEO and TSEO were more closely related than the TCEO. In fact, the chemical composition of TCEO included chemical compounds like p-cymene or γ-terpinene that were not identified in the other two botanical species, as well as trace amounts of other bioactive molecules (Table 3). As a result, the heatmap diagram allowed these species to be clustered based on their chemical composition and the concentration of aromatic components present.

**Table 3:** *Salmonella enterica* strains under this study.

Serovar	Strain	Origin
*S.* Enteritidis	ATTC 13076	Type strain
*S.* Typhi	116	Meat product (sausage)
*S.* Typhimurium	117	Meat product (sausage)
*S.* Typhimurium	686	Pig carcass
*S.* Typhimurium	785	Slaughtering environments
*S.* Enteritidis	217	Meat product (sausage)
*S.* Enteritidis	208	Meat product (sausage)
Monophasic var. *S.* Typhimurium	787	Slaughtering environments
Monophasic var. *S.* Typhimurium	791	Slaughtering environments
Monophasic var. *S.* Typhimurium	792	Slaughtering environments
*S.* Rissen	788	Slaughtering environments
*S.* Rissen	793	Pig carcass

The PCA biplot (Figure 2) showed that the two principal components justified 100% of the total variance, with the first principal component (F1) corresponding to 51.55% and the second principal component (F2) contributing with 48.45% of the total variance. The loading plot exhibited the discrimination of the three EOs along F2, displaying a clear separation. On the contrary, the *S. enterica* strains susceptibility in terms of MICs and the volatile fraction compounds of EOs in the plot were distributed along F1. The biplot revealed the presence of different groups, mainly scattered into three clusters closely related to the three EOs. In Figure 2, when there is a positive correlation between a variable and a treatment, high values are recorded. This means that the volatile compound is present in higher amounts and the EO has higher MICs on the strains recorded closely. Therefore, it was shown that the chemical compounds in CC, TC, and TS EOs were responsible for distinct antimicrobial effects, whereby TSEO displayed the most remarkable bioactivity in the presence of Tc compounds. In detail, the observation that 7 out of the total 11 strains exhibited a negative correlation further supports this noteworthy finding, as illustrated in Figure 2. These *in vitro* data support the conclusion that these chemicals had a relevant effect on the strains that were susceptible to the treatments.

## 3 Discussion

It has been proved that administering Tc to animals orally alters the gut microbiota, which in turn promotes antibiotic resistance in the gut (Grasboll et al., 2017). Alali et al. (2009), Van Gompel et al. (2020) conducted a recent study that demonstrated a significant correlation between the quantity of Tc present in pork and the prevalence of isolates exhibiting resistance to this antibiotic within fecal samples. Consequently, it is imperative to assess novel approaches to combating antimicrobial resistance. Considering this, the antimicrobial activity exhibited by EOs serves as a potential solution for preventing bacterial contamination in animals and, subsequently, food products, thereby offering an intriguing avenue in the fight against antimicrobial resistance (Zhai et al., 2018; McGregor et al., 2019). Furthermore, studies have shown that EO supplementation in swine feed enhances energy digestibility, offering an additional benefit in the context of animal nutrition (Zhai et al., 2018). Therefore, there is a chance to improve animal health and food safety by using EOs as a weapon to battle antimicrobial resistance. According to Langeveld et al. (2014), EOs work by increasing the permeability of the cell membrane and interfering with the activity of efflux pumps. Numerous studies have demonstrated that EOs can target multiple sites inside bacteria, making it harder for them to defend themselves and making it easier for antibiotics to enter, which causes accumulation of the drugs inside bacterial cells. Accordingly, the antibiotics can also have therapeutic effects by weakening specific targets, such as protein synthesis, as highlighted in the research conducted in 2018 by Owen and Laird. Given these unique characteristics, EOs and their individual components have attracted significant interest as a promising alternative to antibiotics in the management of bacterial infections in livestock production, as emphasized by Zhang et al. (2022). Notably, animals raised for food production are a major source of antibiotic resistance genes (ARGs), which may pass from animals to humans mainly through the ingestion of food products obtained from animals (Wang et al., 2023b). *Salmonella enterica* serovar Typhimurium, Enteritidis, and Derby were the primary serovars implicated in human infections, where the Typhimurium serovar exhibited a cosmopolitan distribution, being reported across all four evaluated matrices and continents, and indicating the complexities associated with controlling this pathogen, as it can be transmitted to humans through various routes (Wang et al., 2023b; Ferrari et al., 2019). Therefore, investigating EOs and their derivatives in the animal production chain holds an immense potential to stop the spread of ARGs and reduce the health risks that go along with it. Even though only a few bacterial strains were examined in this study (Table 1), interesting strain-specific responses to treatments were observed (Tables 1, 2), which could depend on the origins and genetic background of the microorganism. Nevertheless, our data confirm the potent antimicrobial effectiveness of EOs against *S. enterica*. Additionally, the EOs phytocomplex may affect how the Tc molecules interact with it, causing *S. enterica* to react differently, depending on the different antimicrobial compounds present in the EOs, which can act as adjuvants to increase their effectiveness (Owen and Laird, 2018). Here, EOs reduced the growth of *S. enterica* strains at low concentration intervals (0.31–5 μL/μL), where Tc compounds were ineffective when provided at the greatest dose (256 μg/μL), as shown in Table 1. The combination of the antimicrobial compounds produced some interesting results (Table 2), including a significant decrease in the effective dose of Tc against *S. enterica* strains (from 256 to 4 μg/μL), suggesting the restored susceptibility of *S. enterica* to the treatment. In addition, the synergy between Tc and EOs was observed in different cases (Table 2), highlighting the potential to lower the dosage of each chemical compound administered and raise a mixture biological effectiveness against a particular target.

On balance, although carvacrol is the main bioactive component in the EOs under investigation, the FICI results suggest that also the secondary antimicrobial compounds in the EOs might have an impact on the activity demonstrated with Tc compounds (Table 4). It is acknowledged that the primary component contributes significantly to the EOs mode of action, though not exclusively. Here, the powerful antibacterial properties of carvacrol work by destroying and destabilizing the bacterial cellular membrane (Lauteri et al., 2022b). However, when combined with Tc compounds, other bioactive molecules such as *α*-terpinene, *γ*-terpinene, linalool, thymol, and p-cymene, may affect the mechanisms of action, creating synergistic effects (Sousa et al., 2022). For instance, the combination of p-cymene with antibiotics exhibits synergistic effects in managing antibiotic-resistant bacteria. Additionally, p-cymene and carvacrol exhibit a strong synergistic effect against planktonic bacterial cultures (Sousa et al., 2022). P-cymene plays a crucial role in enhancing the transport of carvacrol across the lipid layer of bacterial cells, as it can increase the swelling of bacterial cell membranes, which makes it easier for the compound to get inside the cell (Gholami-Ahangaran et al., 2020). Moreover, thymol, linalool, r-carvone, eugenol, eucalyptol, s-carvone, and borneol have anti-plasmid conjugation potential, and decrease the plasmids transfer (Skalicka-Woźniak et al., 2018). The results reported in this study can be justified by the contribution of the EOs phytocomplex to the mechanisms of action established with other antimicrobial compounds. The heatmap (Figure 1), based on the EOs chemical profiles, displayed a clusterization of TSEO and CCEO. This correlation persisted, even though CCEO predominantly contains carvacrol (Table 4), suggesting a closer relation with TSEO than with TCEO. At the same time, PCA revealed a robust correlation between TSEO and the antimicrobial effectiveness in the presence of Tc, suggesting that the phenolic and terpenoid compounds present only in trace amounts (Table 4; Figure 2 in the black circle) may influence the antimicrobial activity established with Tc, amplifying the effect toward the cells and overcoming the strain-specificity of the response. Previous studies by Aelenei et al. (2019) and Kachur and Suntres (2020) have established the combined activity of phenolic, terpenoid, and Tc constituents. In these studies, the MICs of tetracycline were reduced from 8 to 16-fold, respectively. According to the authors, the bioactive phytocomplex and Tc compounds may work together because of the phytocomplex ability to permeabilize membranes, which increases antibiotic penetration and, in turn, increases the effectiveness of antibiotics inside bacterial cells. As a result, Tc compounds could potentially act reversibly by targeting the 30S ribosomal subunit, inhibiting the binding of aminoacyl tRNA to the ribosome (Laure et al., 2021). Furthermore, terpenoid compounds induce membrane disturbance, leading to the uncontrolled release of cytoplasmic materials (Bhavaniramya et al., 2019; Mączka et al., 2022). This disruption results in instability, thereby allowing the entrance of antimicrobial compounds and similar substances. In fact, terpenoid molecules such as thymol and linalool, contain highly active functional groups with delocalized electrons, thereby augmenting their antimicrobial properties (Bhavaniramya et al., 2019). Thymol affects nitric oxide production and glutathione homeostasis, contributing to the recovery from oxidative stress (Ferrari et al., 2019). Conversely, linalool disrupts the activity of crucial enzymes, including succinate dehydrogenase, pyruvate kinase, ATPase, and respiratory chain dehydrogenase (Guo et al., 2021).

**Table 4:** Chemical composition (μL/mL) of *Coridothymus capitatus* (CC), *Thymus capitatus L.* (TC), and *Thymus serpyllum* (TS) EOs.

Chemical compound	CCEO	TCEO	TSEO
1,8 β-phellandrene cineole	-	3.8	-
1,8-cineole	-	-	3.3
1-octen-3-ol	-	2.8	-
Aromadendrene	-	-	2.7
Borneol	3.1	5.7	4.2
Camphene	-	2.4	-
Carvacrol	769.1	685.5	684
Carvacrol acetate	13.8	-	-
Caryophyllene oxide	33.1	-	-
Cedrenol	0.16	-	-
cis-sabinene hydrate	-	-	4.6
c-thujanol	-	0.7	-
d-cadinene	2.2	-	-
Eugenol	3.2	0.3	-
Geranial	-	0.9	-
Geraniol	-	0.1	-
Iso-carvacrol	-	0.4	-
Limonene	-	2.1	2.5
Linalool	-	15.3	82.2
Octanone	-	0.02	-
p-cymene	-	77.3	52.4
Sabinene	-	-	2.8
Terpinen-4-ol	5.6	6.9	6.2
Terpinolene	-	0.12	-
Thymol	-	6.1	25.9
t-thujanol	-	1.8	-
α-bisabolene	37.3	-	-
α-humulene	-	1	-
α-phellandrene	-	1.9	-
α-pinen	-	9.2	7.6
α-terpinene	-	10.9	11.9
α-terpineol	-	1.2	6.1
α-thujene	-	12.3	10.2
β-bisabolene	32	-	-
β-caryophyllene	27.7	25.7	9.4
β-myrcene	-	18.3	14.2
β-phellandrene	-	-	2.3
β-pinene	-	1.4	-
γ-terpinene	-	67.8	40.2

The table displays the components reported according to their elution order on 5% Diphenyl-95% Dimethylpolysiloxan column, using Perkin Elmer Clarus 500 GC/FID/MS.

The findings of this exploratory study highlight the importance of the phytocomplex composition in determining the antibacterial activity of EOs, alone and in combination with Tc, and points out that the effectiveness of the treatment is strain-dependent. Nevertheless, additional research is needed to confirm the insights obtained from the statistical correlations on a wider scale. Future studies in this direction might open new perspectives in the framework of precision medicine, with novel therapeutical strategies based on the specific infection acquired.

## 4 Materials and methods

### 4.1 Bacterial strains

For this study one reference strain of *Salmonella enterica* serovar Enteritidis and eleven strains of *Salmonella enterica* from various stages of the swine food production chain were selected (see Table 3). The isolates belong to the collection of the Department of Food Inspection at the University of Teramo in Italy. Their phenotypic and genotypic resistance profiles were previously investigated by Lauteri et al. (2022b).

*Salmonella enterica* strains were initially cultivated on agar plates containing Müeller–Hinton (MH) medium (Liofilchem, Roseto degli Abruzzi, Italy) and incubated at a temperature of 37°C overnight. Subsequently, a single colony was introduced into MH broth (Liofilchem, Roseto degli Abruzzi, Italy) and subjected to incubation at a temperature of 37°C for 18 h, to attain early stationary phase fresh cultures. Standardized inocula were prepared, with an optical density of 0.1–0.2 at a wavelength of 620 nm, as determined by measurement using a Lambda bio 20 spectrophotometer (PerkinElmer, Markham, Canada). The inocula were further diluted in Phosphate Buffer Saline (PBS) 50 mM, pH 7.4 and 10 μL/mL Tween 80 (Sigma-Aldrich, Milan, Italy) to reach a cellular load of 10^6^ CFU/mL.

### 4.2 Antimicrobial compounds

The experiments were carried out using commercially available, food-grade *Coridothymus capitatus*, *Thymus capitatus* L., and *Thymus serpyllum* EOs, which were kindly provided by Flora S.r.l. (Pisa, Italy). The chemical composition of the EOs is presented in Table 4. The EOs emulsions were prepared by diluting them to a concentration of 80 μL/mL in PBS. Lyophilized tetracycline (>98%), provided by Sigma-Aldrich (Milan, Italy), was used, and its concentration was adjusted to 1 mg/mL through dilution with distilled water.

### 4.3 Determination of minimum inhibitory concentrations and minimum bactericidal concentrations

The MICs of the EOs and Tc compounds were evaluated by microdilution method, according to the CLSI protocol of 2020 (CLSI Guidelines, 2020). This assessment was conducted in a 96-well microtiter plate provided by Corning Incorporated (Kennebunk, ME, United States), and maintained at a temperature of 37°C for 24 and 48 h. The MIC values were determined as the lowest concentration of antimicrobial compounds that did not cause any red discoloration of 2,3,5-triphenyltetrazolium chloride (Sigma-Aldrich, Milan, Italy), added at a ratio of 1 μL/mL to the growth media. The MBCs were subsequently determined after 24 and 48 h at 37°C by plating out the samples onto MH agar plates. The analyses were conducted in three replicates.

### 4.4 Checkerboard assay

The combination of Tc compounds with EOs was evaluated using the Checkerboard assay (Fratini et al., 2017) at a temperature of 37°C for 48 h. The FICI was determined by applying Equation A.1, which allowed the assessment of the interaction between these two antimicrobial compounds. The FICI value indicated whether the relationship between the EOs and Tc compounds was synergistic (FICI <1.0), indifferent (FICI = 1), commutative (1 < FICI value ≤2) or antagonistic (FICI >2.0).


$$\mathrm{FICI}=FIC_{A}^{*}+FIC_{B}^{**}$$

$$FIC_{A}^{*}=MIC_{A}\;\mathrm{in}\;\mathrm{combination}/MIC_{A}\;\mathrm{alone}$$

$$FIC_{B}^{**}=MIC_{B}\;\mathrm{in}\;\mathrm{combination}/MIC_{B}\;\mathrm{alone}\text{.}$$

### 4.5 Data analysis

The chemical composition of the EOs and the MICs of *S. enterica* strains at 37°C for 48 h were analyzed using PCA. Subsequently, a Heatmap diagram was constructed using the XLSTAT 2014 software (Redmond, WA, United States) to cluster the data. The analyses were conducted in three replicates.

## Data Availability

The raw data supporting the conclusions of this article will be made available by the authors, without undue reservation.
